# Intimate partner violence among women with HIV infection in rural Uganda: critical implications for policy and practice

**DOI:** 10.1186/1472-6874-11-50

**Published:** 2011-11-17

**Authors:** Michael O Osinde, Dan K Kaye, Othman Kakaire

**Affiliations:** 1 Kabale Regional Hospital, Department of Obstetrics and Gynecology, P.O.B ox 7, Kabale, Uganda; 2 Department of Obstetrics and Gynecology, School of Medicine, Makerere University College of Health Sciences, P.O. Box 7072, Kampala, Uganda

## Abstract

**Background:**

Intimate partner violence (IPV) is a major public health problem in Africa and worldwide. HIV infected women face increased IPV risk. We assessed the prevalence and factors associated with IPV among HIV infected women attending HIV care in Kabale hospital, Uganda.

**Methods:**

This cross-sectional study was conducted among 317 HIV infected women attending Kabale regional hospital HIV treatment centre, from March to December 2010. Participants were interviewed using an interviewer-administered questionnaire. Data was collected on socio-demographic variables, social habits, and IPV (using the abuse assessment screen and the Severity of Violence against Women Scale to identify physical, sexual and psychological violence). Characteristics of the participants who reported IPV were compared with those who did not. Multivariate logistic-regression analysis was conducted to analyze factors that were independently associated with IPV.

**Results:**

The mean age of 317 respondents was 29.7 years. Twenty two (6.9%) were adolescents and 233 (73.5%) were married or cohabiting. The mean age of the spouse was 33.0 years.

One hundred and eleven (35.0%) were currently on antiretroviral therapy. Lifetime prevalence of IPV (physical or sexual) was 36.6%. In the preceding 12 months, IPV (any type) was reported by 93 respondents (29.3%). This was physical for 55 (17.6%), and sexual /psychological for 38 (12.1%). On multivariate multinomial logistic regression analysis, there was a significant but inverse association between education level and physical partner violence (adjusted relative risk (ARR) 0.50, confidence limits (95% CI) 0.31-0.82, p-value = 0.007). There was a significant but inverse association between education level of respondent and sexual/psychological violence (ARR 0.47 95%CI (0.25-0.87), p-value = 0.017) Likewise, there was a significant inverse association between the education level of the spouse and psychological/sexual violence (ARR 0.57, 95% CI 0.25-0.90, p-value = 0.018). Use of antiretroviral therapy was associated with increased prevalence of any type of violence (physical, sexual or psychological) with ARR 3.04 (95%CI 1.15-8.45, p-value = 0.032).

**Conclusion:**

Almost one in three women living with HIV had suffered intimate partner violence in the preceding 12 months. Nearly one in five HIV patients reported physical violence, and about one in every seven HIV patients reported sexual/psychological violence. Likewise, women who were taking antiretroviral drugs for HIV treatment were more likely to report any type of intimate partner violence (physical, sexual or psychological). The implication of these findings is that women living with HIV especially those on antiretroviral drugs should be routinely screened for intimate partner violence.

## Background

Intimate partner violence (IPV) is a major public health problem in Africa and worldwide [1-7]. For women, its consequences include low birth weight [6], unwanted pregnancy and induced abortion [7] and death from homicide [8]. Other effects include physical injury or disability, depression [9], post-traumatic stress syndrome [9] and preterm birth [10]. In addition, other sequelae include poor acceptance of services to prevent human immunodeficiency virus (HIV) vertical transmission [11]. IPV has been associated with several risk factors, which include (for the male) having multiple sexual partners [4] and perpetrator alcohol use [2,3,12,13]. For the woman, risk factors for IPV include HIV positive status [3,6,11], low education or low socioeconomic status [2,6], pregnancy [7] and being in a cohabitating relationship [7].

Numerous studies in heterosexual relationships [14-19] found an association between IPV and high rates of risk behaviors (such as multiple sex partners, non-use or inconsistent use of condoms and sexual coercion) and sexually transmitted infections (STIs) such as HIV. There are several explanations for this relationship. Firstly, gender differences in socialization of men and women (in socially defined and socially constructed ways) influence who, where and in what context men and women form sexual partnerships, as well as the overt and covert power relationships involved [14]. Secondly, in some patriarchal societies, men's socialization idolizes traits of strengths and toughness, phenomenal sexual success and clustering of violent, anti-social and risky sexual practices [14], as well as women's submissiveness. In such societies, women's sexuality might pose a threat to the socially accepted norms and behavior, as it challenges men's control over women, and provokes jealousy in the women's spouses [14,15]. Women who follow such socially prescribed norms are at high risk of acquiring HIV infection subsequent to their partners' high-risk behavior puts them at risk of acquiring HIV infection.

Substance abuse is often associated with risky sexual behavior [14-16]. IPV increases women's risk of acquisition of HIV as a result of coercive sex (in case of sexual violence), non-use of condoms and increased risk of exposure to HIV [17-19]. Abusive partners have other risk behaviors such as drinking alcohol, drug abuse, STIs, multiple sexual partners and concurrent sexual partnerships. Abused women face increased HIV risk based both on the greater likelihood of HIV infection among abusive husbands and elevated HIV transmission within abusive relationships [17-19]. IPV affects the psychological and mental health of women survivors, in part through increasing other risk behaviors such as drug and substance abuse [16]. Indeed, alcohol use before sex and related disinihibition were found to increase therisk of HIV infection in young women aged 15-24 years in Uganda [17].

In addition, IPV may be a consequence of HIV care. Where as voluntary counseling and testing (VCT) and routine counseling (RCT) are cornerstones of HIV prevention interventions and are entry points into care for those who test HIV positive, the behaviors promoted after testing positive increase risk of IPV. Such behaviors include negotiating condom use with sexual partners, abstaining from sex and disclosure of HIV status to sexual partners [20]. To overcome this problem, couple counseling has been advanced as a measure to promote HIV prevention and reduce IPV risks. IPV functions as both a risk marker and a risk factor for HIV among women [21]. Indeed, among women attending antenatal clinic in South Africa and Nigeria, a strong association was found between gender-based violence and HIV infection [22-25], suggesting the need for routine HIV and IPV screening for Prevention of Mother to Child HIV transmission (PMTCT) programs. Given that IPV increases women's risk of acquiring HIV and that disclosure of HIV status or negotiating safer sex could be catalysts for gender-based violence [20], integrating gender-based violence screening and management could be a critical component of HIV prevention and care programs. The objective of this cross-sectional study was to assess the prevalence and factors associated with IPV among HIV infected women attending HIV care in Kabale Hospital, Uganda.

## Methods

### Study design, participants and data collection procedure

This cross-sectional study was conducted at Kabale regional hospital, from March to December 2010. During this period, 317 women were recruited into the study from among women who received medical care and counseling services at the HIV treatment centre. Participants were interviewed using an interviewer-administered questionnaire adapted from the demographic and health survey [12]. Data was collected on socio-demographic variables such as age, level of education, marital status, employment status, age of spouse, occupation of spouse and social habits (drinking alcohol or smoking) as well as the education level of spouse. For assessment of IPV, we used the Abuse Assessment Screen by McFarlane et al [26] and the Severity of Violence against Women Scale [27]. We inquired whether the respondents had experienced violence from their spouses over their lifetime or in the preceding 12 months (separating physical, sexual and psychological violence. We obtained ethical approval from Kabale Hospital and Mbarara University Ethics committees. Counseling about IPV was provided to all the participants, and all eligible participants were provided with appropriate counseling, support and antiretroviral therapy.

### Data analysis

Data was analyzed by computing frequencies and percentages for categorical variables as well as means and standard deviations for numerical variables, stratified for presence or absence of IPV. On bivariate analysis, factors associated with IPV were analyzed, using chi-square test for categorical variables and Student's *t*-test for numerical variables, at the 95% significance level. Multivariate multinomial logistic-regression analysis was conducted to analyze factors that were independently associated with IPV, computing relative risks for IPV. During the stepwise multinomial logistic regression modeling, IPV in the preceding 12 months was coded at three levels: any type of violence, physical violence only and sexual/psychological violence. Absence of IPV was the reference category.

## Results

Regarding socio-demographic characteristics, the mean age of the 317 women was 29.7 years (standard deviation 9.9 years). Twenty two (6.9%) were adolescents while 233 (73.5%) were married or cohabiting. The mean age of the spouse was 33.0 years (standard deviation 8.6 years). The respondents were of high parity with 164 women (51.7%) being of parity 5 or higher. Regarding education level, 184 women (58.5%) had just primary level or no formal education. Lifetime prevalence of IPV (physical or sexual) was reported by 116 (36.6%) respondents. In the preceding 12 months, IPV (any type) was reported by 93 respondents (29.3%). This was physical for 55 (17.6%), and sexual/psychological for 38 (12.1%). Table 1 shows the socio-demographic characteristics of the respondents stratified by presence of IPV in the preceding 12 months. The respondents who reported history of IPV were comparable to those who did not (p > 0.05) with respect to all socio-demographic variables except level of education (of the respondents) (p-value = 0.002) and level of education of their spouses (p-value = 0.037).

**Table 1:** Socio-demographic characteristics of the respondents stratified for presence or absence of intimate partner violence in the preceding 12 months

Characteristic	All participants Number (percentage)	History of IPV Number (percentage)	No history of IPV Number (percentage)
*Age group of respondent*			
24 years or less	125 (39.4)	22 (40.0)	103 (39.3)
Above 24 years	192 (60.6)	33 (60.0)	159 (60.7)
*Religion of respondent*			
Catholic	120 (38.0)	14 (25.5)	106 (40.5)
Protestant	168 (53.2)	28 (50.9)	130 (49.6)
Moslem	16 (5.1)	2 (3.6)	14 (5.3)
Others	12 (3.8)	1 (1.8)	11 (4.2)
*Education level of respondent			
No formal education	27 (8.5)	7 (12.7)	20 (7.6)
Primary level	157 (50.0)	35 (63.6)	123 (46.9)
Secondary level	81 (25.6)	11 (20.0)	70 (26.7)
University or higher than secondary	50 (15.8)	2 (3.6)	48 (18.3)
*Drinking*			
Yes	34 (10.8)	6 (10.9)	28 (10.7)
No	283 (89.2)	49 (89.1)	233 (89.3)
*Parity*			
1	52 (16.4)	8 (14.5)	44 (16.8)
4-Feb	101 (31.9)	21 (38.2)	80 (30.5)
5 and above	164 (51.7)	26 (47.3)	138 (50.7)
*Marital status of respondent*			
Single	23 (7.3)	2 (3.6)	21 (8.0)
Currently married	227 (71.6)	48 (87.4)	179 (68.3)
Ever married	67 (21.1)	5 (9.0)	62 (25.7)
*Type of marriage if currently married*			
Monogamous	200 (88.2)	48 (87.3)	179 (74.3)
Polygamous	117 (11.8)	5 (12.7)	62 (25.7)
*Education level of spouse			
No formal education	9 (3.6)	3 (5.5)	6 (2.3)
Primary level	96 (41.0)	23 (41.8)	73 (27.8)
Secondary level	92 (39.3)	19 (48.2)	73 (27.8)
University or higher than secondary	37 (15.8)	3 (5.5)	34 (42.1)

Key: * The respondents who reported history of IPV in the preceding 12 months were comparable to those who did not (p > 0.05) except level of education (of the respondents) (p-value = 0.002) and level of education of their spouses (p-value = 0.037).

Table 2 shows the respondents' treatment with antiretroviral therapy (ART) in relation to the prevalence of intimate partner violence in the preceding12 months. One hundred and eleven women (35.0%) were currently on ART. The mean duration of ART was 3.3 months (± 3.2 months). Thirty four women (30.6%) had changed their treatment regimen to second line regimen, with side effects of the drugs being the main reasons for treatment regimen change. (The commonest ART first line regimen was Triomune, a fixed dose combination of Stavudine, Lamivudine and Nevirapine, which frequently caused side effects and was eventually phased out from the available treatment regimes). Women who were currently on ART were nearly twice as likely to be report IPV in the preceding 12 months (Odds ratio 1.9, 95% CI 0.93-3.93), p-value = 0.078).

**Table 2:** Drug therapy and Intimate partner violence in the preceding 12 months

Characteristic	All respondents	History of IPV	No History of IPV
	Number (Percentage)	Number (Percentage)	Number (Percentage)
*Are you currently on Antiretroviral therapy*			
Yes	111 (35.0)	13 (23.6)	98 (37.4)
No	206 (65.0)	42 (76.4)	164 (62.6)
*Have you ever changed Antiretroviral treatment regimen*			
Yes	34 (30.6)	7 (63.6)	27 (27.6
No	77 (69.4)	4 (37.3)	71 (72.3)
*Reasons for changing ARV treatment regimen*			
Side effects of drugs	20 (58.8)	2 (33.3)	18 (64.3)
Drug resistance (treatment failure)	7 (20.6)	2 (33.3)	5 (17.9)
Pregnancy-related	3 (8.8)	1 (16.7)	2 (7.2)
Reasons Others (drug costs, Tuberculosis co-infection)	4 (11.8)	1 (16.7)	3 (10.7)

Table 3 shows the results of multivariate multinomial logistic regression analysis showing factors associated with IPV in the preceding 12 months. Factors associated with lower prevalence of physical violence were education level of respondent (adjusted relative risk (ARR) 0.50, confidence limits (95%CI) 0.31-0.82), education level of spouse (ARR 0.5, 95%CI 0.31-0.82, p = 007) and use of antiretroviral therapy (ARR 2.56, 95%CI 0.98-7.32, p-value = 0.054). There was an inverse association between age of respondent (ARR 0.93, 95%CI 0.87-1.08, p-value = 0.054), education level of respondent (ARR 0.47, 95%CI 0.25-0.87, p-value = 0.017) and education level the spouse of respondent (ARR 0.47, 95%CI 0.25-0.90, p-value = 0.018) and presence of sexual/psychological violence. Only use of ART was significantly associated with prevalence of any type of violence (physical, sexual or psychological) with ARR 3.04 (95%CI 1.15-8.45, p-value = 0.032), after adjusting for age, parity, education level and employment status of the respondent.

**Table 3:** Multinomial multivariate logistic regression of factors associated with Intimate partner violence in the preceding 12 months

Characteristic	Adjusted relative risks	p-value
	With confidence limits	
**Physical violence**		
Education level of respondent (secondary or higher versus primary level or no formal education)	0.50 (0.31-0.82)	0.007
Use of anti retroviral drugs (ARVs) Yes versus No	2.56 (0.98-7.32)	0.054
**Sexual and psychological violence**	0.93 (0.87-1.08)	0.054
Age of respondent		
Education level of respondent (secondary or higher versus primary level or no formal education)	0.47 (0.25-0.87)	0.017
Education level of Spouse (secondary or higher versus primary level or no formal education)	0.47 (0.25-0.90)	0.018
Use of anti retroviral drugs (ARVs) Yes versus No	2.58 (1.19-6.17)	0.03
**Any type of Violence**		
Use of anti retroviral drugs (ARVs) Yes versus No	3.04 (1.15-8.45)	0.032

## Discussion

Violence against women has been identified as a major risk factor for HIV infection among women [28-31]. Observational studies have shown that a woman's exposure to IPV is associated with an increased risk for HIV infection in southern and eastern Africa [3,4,13-15,21,22,32]. There are several mechanisms through which IPV is related to increased risk for HIV infection among women. These include direct effects through higher levels of violent sexual intercourse among abused women. This is in addition to the likelihood that men who enact IPV will have more high-risk sexual behaviors [33,34] and may be more likely to be HIV infected [23]. Furthermore, there are indirect effects through reduced likelihood that women who have suffered past abuse are likely to have more sexual partners and more transactional sex, as well as being less likely to test/disclose sero-status or less receptive to HIV awareness programs [35,36]. These reflect an underlying power imbalance. Indeed, two cohort studies in South Africa have found reduced HIV risk behaviors [37] and reduced HIV incidence [38] following interventions to empower women.

In our study, there was an inverse association between education level of spouse with sexual/psychological violence. The explanation for this is unclear. Where as a woman's education level may reduce gender inequality and gender-power imbalance in some contexts, it may increase insecurity in the male partners and become one of the contextual factors for increased IPV in other contexts [14-16]. In addition, gender inequality and polygamy have been associated with increased risk of IPV. Likewise, there is no evidence that the more educated the woman is, the less likely they will be in polygamous marital relationships. There is no evidence that marital conflict, drug abuse or excessive alcoholic consumption occur with lower frequency in sexual relationships of more educated women. However, women in such relationships may have more bargaining power and potential to leave such abusive relationships. In the latter case, such leaving or threats of leaving may trigger intimate partner violence.

In this study, we acknowledge several limitations: this was a cross-sectional study in women attending a health facility with a regional HIV care centre, where we evaluated only 317 respondents, all of whom were HIV positive. Our findings are not representative of all HIV patients or all survivors of IPV. Since the respondents were women already accessing HIV care, it is possible that they experienced lower levels of violence that the HIV infected women in the general population. Secondly, we can not establish a temporal relationship between HIV and IPV. Thirdly, since we have no reference group that was HIV negative, we can not evaluate a causal relationship between IPV and HIV. Our findings, however, suggest that IPV is associated with HIV infection, as almost every third woman living with HIV had suffered IPV, and about one in seven HIV patients reported both sexual and psychological violence in the preceding 12 months.

Our findings are in agreement with studies from Tanzania [4], Rwanda [39] and South Africa [40,41] which found an association between HIV and IPV. The high prevalence of IPV (both physical and sexual violence) in women with HIV infection has critical implications for policy and practice in programs and interventions for HIV or IPV. Such programs/interventions should incorporate strategies to address the problem on several fronts. Firstly, the strategies should change the underlying social construction of masculinity and gender power imbalances that underlie risk for IPV and HIV. The experience of violence reinforces gendered power inequalities that impact on women's HIV risk, such that women who have less power in their sexual relationship (measured on the Sexual Relationship Power Scale) are at higher risk of having HIV [13] and women with less power have a lower likelihood of condom use [14,41]. In addition, health education should be provided to women receiving care for HIV or IPV regarding the nature of the associated risk, as well as reduction of risk factors (that increase risk of both HIV and IPV).

Secondly, policy guidelines should incorporate routine screening for IPV and HIV in healthcare settings. Thirdly, clinicians should incorporate inquiries about IPV to women accessing care for STIs or HIV. Similarly, STIs should be recognized as potential epidemiological markers for IPV. Lastly, our findings have implications for future research: A larger study comprised of both HIV positive and HIV negative participants would confirm whether a true association exists between HIV infection and IPV. Furthermore, though their findings tend to be context-specific and their generalisability is limited, qualitative studies should be conducted in this population and setting to develop more nuanced understanding of the complex interaction between gender norms, IPV and HIV. In addition, association between use of antiretroviral therapy and IPV requires further investigation in longitudinal community based studies, given its grave possible repercussions at the personal and public health levels, resulting from viral resistance that could develop in case of poor adherence to medication.

## Conclusion

Nearly one in five women living with HIV had suffered IPV in the preceding 12 months, and one in every six HIV patients with sexual violence reported psychological violence. The higher the education level attained by the respondent (or their spouse), the less likely they were to report sexual or psychological violence. Likewise, women who were taking antiretroviral drugs for HIV treatment were more likely to report any type of IPV. The implication of these findings is that women living with HIV especially those on antiretroviral drugs should be routinely screened for intimate partner violence

## Competing interests

The authors declare that they have no competing interests.

## Authors' contributions

DKK and OK conceptualized the study. DKK, OK and MOO designed the study instrument. MOO piloted the study instruments and collected the data. DKK and OK conducted the data analysis. DKK wrote the first draft of the manuscript. All co-authors contributed to revision of the subsequent drafts and approved the final version of the manuscript.

## Pre-publication history

The pre-publication history for this paper can be accessed here:

http://www.biomedcentral.com/1472-6874/11/50/prepub
